# Investigating Inherited Heart Diseases Using Human Induced Pluripotent Stem Cell-Based Models

**DOI:** 10.3390/life14111370

**Published:** 2024-10-25

**Authors:** Brian Xiangzhi Wang

**Affiliations:** Department of Cardiology, Jersey General Hospital, Gloucester Street, St. Helier JE1 3QS, Jersey, UK; brian.wang15@imperial.ac.uk

**Keywords:** inherited heart disease, stem cell, disease modelling, arrhythmia, tissue engineering

## Abstract

Inherited heart diseases (IHDs) are caused by genetic mutations that disrupt the physiological structure and function of the heart. Understanding the mechanisms behind these diseases is crucial for developing personalised interventions in cardiovascular medicine. Development of induced pluripotent stem cells, which can then be differentiated to any nucleated adult cell type, has enabled the creation of personalised single-cell and multicellular models, providing unprecedented insights into the pathophysiology of IHDs. This review provides a comprehensive overview of recent advancements in human iPSC models used to dissect the molecular and genetic underpinnings of common IHDs. We examine multicellular models and tissue engineering approaches, such as cardiac organoids, engineered heart tissue, and multicellular co-culture systems, which simulate complex intercellular interactions within heart tissue. Recent advancements in stem cell models offer a more physiologically relevant platform to study disease mechanisms, enabling researchers to observe cellular interactions, study disease progression, and identify therapeutic strategies. By leveraging these innovative models, we can gain deeper insights into the molecular and cellular mechanisms underlying IHDs, ultimately paving the way for more effective diagnostic and therapeutic strategies.

## 1. Introduction

### 1.1. Global Health Burden of Inherited Heart Diseases

Inherited Heart Diseases (IHDs) pose a significant and growing burden on global public health. Conditions such as hypertrophic cardiomyopathy (HCM), arrhythmogenic right ventricular cardiomyopathy (ARVC), and long QT syndrome (LQTS), contribute to severe cardiac complications, including heart failure, arrhythmias, and sudden cardiac death. Recent global estimates suggest that inherited heart diseases affect approximately 1 in 500 individuals, with HCM alone being responsible for 36% of all cases of sudden cardiac death in young people [1,2,3,4]. The prevalence of IHDs is increasing due to improved diagnostic capabilities, but this rise further amplifies the pressure on healthcare systems, especially in resource-limited settings.

From an economic perspective, IHDs impose a significant financial strain on both healthcare systems and affected families. The cost of long-term management, ranging from genetic testing and diagnostic evaluations to ongoing treatments like medications, implantable cardioverter defibrillators (ICDs), and heart transplants, contributes to the overall economic burden. Moreover, the indirect costs related to lost productivity, disability, and the psychosocial impact on families are considerable, further highlighting the need for targeted research and intervention strategies. A 2022 report estimated that the annual direct and indirect costs of cardiovascular disease in the United States alone reached USD 378 billion, including USD 226 billion in direct healthcare expenditures and USD 152 billion in indirect costs such as lost productivity, illustrating the far-reaching economic impact of these conditions [5].

Addressing the global health burden of IHDs requires a comprehensive approach that includes raising awareness, improving diagnostic accuracy, expanding access to genetic testing, and advancing research into effective treatments. Given the growing number of affected individuals and the economic burden, prioritising IHD research and improving patient care has never been more urgent.

### 1.2. Genetic Basis and Pathophysiology of IHDs

The genetic basis of IHDs is complex and involves mutations in various genes that encode proteins crucial for cardiac function. These mutations can lead to structural abnormalities, impaired cardiac muscle function, or electrical disturbances within the heart. For instance, HCM is often caused by mutations in genes that encode sarcomeric proteins, leading to abnormal thickening of the heart muscle [6]. Similarly, LQTS, a condition that predisposes individuals to life-threatening arrhythmias, is typically linked to mutations in genes responsible for ion channel function [7]. In recent years, advancements in genetic sequencing has led to the discovery of novel genetic mutations linked to IHDs, further expanding our understanding of the molecular basis of these diseases. For instance, recent studies have identified mutations in genes like *FLNC*, associated with arrhythmogenic cardiomyopathy, and *TTN*, which plays a significant role in dilated cardiomyopathy [8,9,10,11]. These findings offer new insights into disease pathogenesis and open potential avenues for targeted therapies.

Gene therapy has emerged as a promising approach for treating inherited cardiac conditions, offering the potential to correct disease-causing mutations at their source. In the context of IHDs, gene therapy developments have made significant strides, particularly in the repair or silencing of defective genes through techniques such as CPRISPR-Cas9 and RNA-based therapies. Although these therapies are still in the experimental phase, preclinical studies have shown encouraging results, particularly in diseases like LQTS and HCM, where targeted interventions could prevent or mitigate disease progression. These advances represent an exciting frontier in the treatment of IHDs, with the potential to offer curative therapies rather than lifelong symptom management.

Moreover, in clinical practice, the presentation of inherited arrhythmias, especially in paediatric populations, is a significant concern. Recent work has highlighted the unique challenges faced by children with inherited arrhythmias, including the difficulties in early diagnosis and management [12]. This review emphasises the need for heightened clinical vigilance in paediatric cases, as children with inherited arrhythmias often present with more severe symptoms and may require different therapeutic approaches compared to adults. Incorporating such findings into clinical practice can greatly enhance our understanding and treatment of IHDs across different age groups.

The pathophysiology of IHDs varies depending on the specific genetic mutation and its impact on cardiac tissue. In many cases, the disease process begins at the molecular level, where defective proteins disrupt normal cardiac function, leading to progressive structural changes, impaired contractility, or altered electrical activity [13,14,15]. Understanding these genetic and pathophysiological mechanisms is crucial for developing targeted therapies and improving outcomes for patients with IHDs.

### 1.3. Challenges in Understanding IHD Mechanisms

Despite significant advances in the study of IHDs, numerous challenges remain in fully understanding their underlying mechanisms. One major challenge is the genetic heterogeneity of these diseases; mutations in different genes can result in similar clinical phenotypes, complicating diagnosis and treatment. Additionally, the penetrance and expressivity of genetic mutations that lead to IHDs can vary widely, meaning that individuals with the same mutation may experience vastly different symptoms, making it difficult to predict disease course and outcomes [16]. Another challenge lies in the complexity of the heart as an organ, where multiple cell types, signalling pathways, and structural components interact in ways that are not yet fully understood. Traditional animal models often fail to capture the full spectrum of human IHDs, and while advances in genetic technology have improved our ability to study these diseases, translating these findings into effective therapies remains a formidable task [17]. Moreover, ethical and practical limitations in obtaining human heart tissue for study hinder progress [18]. As a result, there is an ongoing need for innovative research approaches, including the use of stem cells and advanced imaging techniques, to better understand the mechanisms driving IHDs and develop more effective treatments.

### 1.4. Advances in Stem Cell Technology for IHD Research

Stem cell technology has revolutionised research into IHDs, offering new insights into disease mechanisms and potential therapeutic avenues. One of the most promising developments is the use of induced pluripotent stem cells (iPSCs), which can be generated from a patient’s own cells and differentiated into cardiomyocytes or other cell types that make up heart muscle. These patient-specific iPSCs allow researchers to model IHDs in vitro, studying the effects of genetic mutations on heart cells and identifying potential drug targets. Furthermore, human iPSC-derived cardiomyocytes (iPSC-CMs) can be used to screen for drug efficacy and toxicity, paving the way for personalised medicine approaches in the treatment of IHDs. Recent advances have also seen the development of three-dimensional cardiac tissue models, or “heart-on-a-chip” systems, which more accurately replicate the complex structure and function of the human heart [19]. These models are invaluable for studying the pathophysiology of IHDs and testing new therapeutic interventions in a controlled environment. As stem cell technology continues to evolve, it holds great promise not only for understanding the molecular mechanisms of IHDs but also for developing innovative treatments that could one day repair or replace damaged heart tissue in affected patients.

## 2. Stem Cell-Derived Models

### 2.1. Historical Overview: From hESCs to iPSCs

Unicellular stem cell models enable a focused and controlled environment to study the intricacies of cellular function and dysfunction in IHDs. These models are particularly useful in investigating the molecular and genetic underpinnings of IHDs at a cellular level, offering insights that are often difficult to obtain from more complex multicellular models. The first human stem cell-derived cell types were developed from human embryonic stem cells (ESCs) over 25 years ago [20,21,22]. The genes that encode transcription factors pivotal in the maintenance of pluripotency are Oct4, Sox2, and Nanog [23,24]. However, unless the embryo for the human ESCs carried a specific, highly penetrant disease mutation, human ESC-derived cell types had poor predictive value. Furthermore, the sourcing of these cells had major ethical issues, limiting their use [25]. The field then moved to exploring alternative patient-specific stem cells.

### 2.2. Human iPSCs

#### 2.2.1. Generation of Patient-Specific iPSCs

A more recent approach for producing stem cell-derived cell types, including cardiomyocytes, came following the production of iPSCs [26]. These cells are created by reprogramming somatic cells back to a pluripotent state. High expression of four specific transcription factors, Oct4/Sox2/Klf4/c-Myc or Oct4/Sox2/Nanog/LIN28 by virus, can reprogram fibroblast cells into an embryonic-like state [26,27]. Since then, alternative methods of reprogramming have been shown, such as generating iPSCs from fibroblasts by viral integration of Oct4/Sox2/Klf4 without c-Myc [28]. These cells are then capable of differentiating into any cell type, including cardiomyocytes. However, this original method, by relying on viral integration, posed concerns regarding potential genomic alterations and tumorigenicity.

To address these limitations, current studies have shifted toward non-integrating reprogramming methods, such as Sendai viral vectors, which are episomal and do not integrate into the host genome, as well as mRNA-based expression systems that transiently express the necessary reprogramming factors without altering the host DNA [29,30,31]. These non-integrating approaches have significantly improved the safety of reliability of iPSC generation, making them more suitable for clinical applications.

In addition to the use of fibroblasts, a variety of other somatic cell types have been successfully reprogrammed into iPSCs. Cells derived from blood (T lymphocytes), urine, and even hair follicle keratinocytes have been used as starting materials for generating iPSCs [32,33,34]. These alternative sources provide a less invasive option for obtaining patient-specific cells, particularly in clinical and disease-specific applications. The use of these different cell types expands the accessibility of iPSC technologies and further promotes the development of personalised disease models for investigating IHDs.

Since the first production of iPSCs, they have been differentiated into various cell types found in the adult myocardium, including cardiomyocytes, endothelial cells, smooth muscle cells, and others (Table 1). These patient-specific iPSCs allow researchers to model disease-specific phenotypes, test drug efficacy, and explore potential gene-editing strategies for treating inherited conditions.

#### 2.2.2. Differentiation of iPSCs into Cardiomyocytes

Various protocols have been established to promote differentiation of iPSCs into cardiomyocytes. These protocols are discussed in detail elsewhere [58]. The most efficient method to generate iPSC-CMs has a two-stage approach, in which iPSCs are treated with a Glycogen Synthase Kinase (GSK) inhibitor to induce the beta-catenin pathway which drives conversion of iPSCs into cardiac progenitor cells [59]. These cells are then cultured in the presence of broad-spectrum pharmacological agents which target Wnt pathways, producing a phenotype that can contract and respond to physiological and pharmacological stimuli in a manner that is comparable to the adult, human cardiomyocyte.

#### 2.2.3. Advantages of Human iPSC Models for IHD Research

The use of human iPSC models to investigate IHDs allows a personalised model that reflects the patient’s unique genetic background. To date, human iPSC models have been generated on a wide range of channelopathies, cardiomyopathies and vascular diseases [60]. Furthermore, they allow researchers to observe the development and progression of IHDs from the early stages, providing valuable insight into the initial cellular and molecular events critical to the development of IHDs. Virtually all models used for drug screening and disease modelling rely on the use of cardiomyocytes from animal models or isolated human adult cardiomyocytes [61,62]. There are many challenges with the use of current human and animal models, including high costs, difficulties in manipulation, and ethical issues [61,62]. Importantly, human iPSCs hold significant value in drug screening, as differentiated cells developed from iPSCs can be used to test the efficacy and safety of novel drugs, enabling personalised medicine approaches.

### 2.3. Studying Cellular Function and Dysfunction

#### 2.3.1. Electrophysiology Studies

Researchers can use human iPSC-CMs to investigate how genetic mutations associated with IHDs affect cellular function. From identifying the first genetic mutation causing an arrhythmia in 1995, there are now over 28 genes implicated in inherited cardiac arrhythmic disorders [63,64]. Electrophysiology studies allow researchers to investigate how mutations alter the electrical properties of cardiomyocytes, leading to arrhythmias and other electrical disturbances associated with IHDs. LQTS type 1 (LQTS1) was the first ion channelopathy modelled using human iPSC-CMs. In a study by Moretti and colleagues, human iPSCs were derived from two LQT1 patients with heterozygous missense mutation R190Q in the gene *KCNQ1* [44]. The human iPSC-CMs created from these cells demonstrated the reduction in action potential repolarising current *I_Ks_* identified in adults with this condition. The resulting prolongation of the action potential duration puts these patients at risk of arrhythmic events when exposed to isoproterenol, a beta-adrenergic agonist. Homozygous mutations in *KCNQ1* has been shown to cause Jervell and Lange-Nielsen Syndrome (JLNS), an autosomal recessive disorder characterised by a prominent prolongation of the QT interval and deafness [65]. Human iPSC-CMs derived from patients with JLNS or genetically engineered to have a homozygous mutation in *KCNQ1* demonstrate the expected pronounced action potential duration prolongation and susceptibility to drug-induced arrhythmia [66].

#### 2.3.2. Contractility Studies

Contractility studies involving the analysis of how genetic defects impact the contractile function of cardiomyocytes are important for investigations into conditions like HCM and dilated cardiomyopathy (DCM). Mutations that truncate the sarcomere protein titin are the most common genetic cause for DCM [67]. Hinson and colleagues used human iPSC-CMs to identify that missense mutations cause truncations in the A-band domain of titin that cause DCM. Interestingly, truncation in the I-band is better tolerated [68].

#### 2.3.3. Calcium Handling Studies

Calcium handling studies investigate how mutations affect the cycling of calcium within cardiomyocytes, a process that is crucial for physiological contraction and relaxation of heart muscle [69]. El-Brattrawy and colleagues investigated the pathogenesis of patients with Short QT-Syndrome (SQTS) [70]. They generated human iPSC-CMs from the skin fibroblasts of a patient with SQTS type 1 carrying a mutation (N588K) in *KCNH2* and two healthy control subjects. This mutation increased the expression of HERG channels and altered calcium homeostasis in the human iPSC-CMs, contributing to a greater frequency of irregular, delayed afterdepolarisation-like and early afterdepolarisation-like events than control.

### 2.4. Genome Editing in iPSC-CMs

#### 2.4.1. CRISPR/Cas9 Technology

The advent of CRISPR/Cas9 genome editing has revolutionised the ability to study genetic diseases. This technology allows precise modification of specific genes within iPSCs, enabling researchers to introduce or correct genetic mutations associated with IHDs [71,72]. This system introduces double-strand breaks (DSBs) at specific sites in the genome, which are then repaired endogenously by the cell. This is often by error-prone nonhomologous end-joining (NHEJ), which often results in insertion or deletions (indels) at the repair site.

However, when an exogenous donor template with homology to the sequences flanking the DSB is provided, the cell can use the homology-directed repair (HDR) pathway [73]. This pathway allows for precise modifications, ranging from the introduction of single-nucleotide changes to the insertion of multi-kilobase sequences into the genome [74]. HDR is particularly useful for modelling specific disease-causing mutations or for introducing therapeutic corrections in iPSCs.

By using CRISPR/Cas9 in conjugation with HDR, researchers can generate isogenic cell lines, where only the gene of interest differs between the experimental and control groups [75]. This approach, which typically takes 2–6 months, eliminates background genetic variability, allowing for a clearer understanding of the specific effects of a mutation and enabling the development of targeted therapies.

#### 2.4.2. Knockout and Knock-In Models

Gene editing technologies have been utilised in studying the function of genes involved in the pathophysiology of IHDs. Knockout models disable specific genes in iPSC-CMs to study their role in heart function and disease. Knock-in models introduce specific mutations associated with IHDs to replicate the disease phenotype and study its mechanisms. Human iPSCs have been derived from a patient with LQTS and confirmed to carry a novel variant of uncertain significance (VUS) in *KCNH2* [76]. Upon correcting the VUS with a knockout model, the electrophysiological abnormalities observed in the patient human iPSC-CMs were corrected, and introducing the homozygous variant into a control human iPSC line by knock-in enabled researchers to confirm its contribution to electrophysiological dysfunction.

## 3. Multicellular Models and Tissue Engineering

Multicellular models and tissue engineering approaches offer a more comprehensive and physiologically relevant platform than single-cell models to study the mechanisms of IHDs. These models simulate the complex interactions between different cell types within the heart, providing deeper insights into disease pathophysiology [77,78,79]. Recent advancements in the field have led to the creation of sophisticated models that more accurately mimic human heart tissue in both structure and function. These include cardiac organoids, engineered heart tissue (EHT), and co-culture systems that are increasingly being used to develop and test therapeutic strategies in a controlled environment. Such systems are proving invaluable for understanding not only disease mechanisms but also for advancing personalised medicine through the screening of drug efficacy and potential gene therapy interventions.

### 3.1. Cardiac Organoids

#### 3.1.1. Structure and Function of Cardiac Organoids

The human heart is primarily composed of cardiomyocytes (60%), with the predominant non-myocyte cell types being cardiac fibroblasts (24%) and endothelial cells (14%). The rest of the heart consists of a small number of smooth muscle cells, epicardial cells, conductance cells as well as immune cells [80,81]. Cardiac organoids are three-dimensional structures that replicate aspects of heart tissue architecture and function. These organoids are derived from stem cells, typically iPSCs, which are induced to differentiate into various cardiac cell types, including cardiomyocytes, fibroblasts, and endothelial cells. Organoids can be generated through self-organisation or by guiding the differentiation process using specific growth factors and signalling molecules [82,83].

Recent developments have significantly enhanced the functional complexity of cardiac organoids, enabling the study of multiple cell types interacting within a tissue-mimetic environment. This makes them highly valuable for studying the events in the pathogenesis of IHDs. Furthermore, cardiac organoids offer a platform for high-throughput screening of potential drugs and therapeutic agents, allowing researchers to assess their efficacy and safety in a more physiologically relevant context. These advancements underscore the translational potential of organoids for drug discovery and therapeutic testing, providing an essential tool for personalised medicine approaches.

#### 3.1.2. Applications in IHD Research

Researchers have been able to perform mutagenic studies by using iPSCs derived from patient tissue to create cardiac organoids physiologically relevant to accurately model familial cardiomyopathy in vitro. Yang and colleagues created cardiac organoid constructs using human iPSCs and human marrow stromal cells from patients with a myosin heavy-chain 7 mutation [*E848G*]. The resulting organoid structures demonstrated decreased alignment and reduced systolic function with little impact on diastolic function, recapitulating the contractile dysfunction in these patients. They were able to demonstrate that the E848G allele disrupts the protein–protein interaction between the MYH7 and cardiac myosin-binding protein C, providing new targets for therapeutic interventions [84].

#### 3.1.3. Case Studies: Modelling Cardiomyopathies and DMD

Similarly, Buono and colleagues used human iPSC-CMs derived from a HCM patient with an R719Q mutation in *MYH7*, a single point mutation that has been known to play a role in the onset of HCM causing sudden cardiac death [85,86]. They combined these human iPSC-CMs with human cardiac endothelial cells and cardiac fibroblasts to create an organoid that exhibited an arrhythmogenic phenotype [86]. This can then play a critical role in drug screening for therapeutic strategies against sudden cardiac death in HCM patients.

Recent advancements in the application of iPSCs in disease modelling have also been demonstrated in research related to Duchenne Muscular Dystrophy (DMD). Cardiac organoids have been constructed using cardiomyocytes derived from patients with DMD [87,88]. This disease affects approximately 1 in 5000 males and is caused by mutations in the X-linked dystrophin gene (*DMD*) [89]. The dystrophin protein links the cytoskeleton and extracellular matrix of muscle cells, as well as maintaining the integrity of the plasma membrane [90]. In DMD, the dystrophin protein is absent or dysfunctional, causing muscle degeneration and often DCM. By creating organoids where the DMD mutation was corrected using CRISPR/Cas9 technology, the researchers were able to restore dystrophin expression and improve the contractile ability of the tissue. These studies highlight the value of human cardiac organoids for modelling IHDs and demonstrate the functional impact that gene editing can have on human cardiac tissue.

In a recent study, Eguchi and colleagues explored the role of telomere shortening in cardiomyocytes affected by DMD and investigated the potential of telomere preservation as a therapeutic approach. The researchers utilised iPSCs derived from DMD patients to generate cardiomyocytes and compared these DMD iPSC-CMs to control cells. The study found that DMD iPSC-CMs exhibited reduced cell size, nuclear size, and sarcomere density compared to controls on day 30 of differentiation. To address telomere attrition, the researchers focused on the overexpression of telomeric repeat-binding factor 2 (TRF2), a key component of the Shelterin complex, and found that TRF2 expression rescued deficiencies in cell size and sarcomere density.

In addition to improving cellular morphology, TRF2 overexpression ameliorated the activation of the DNA damage response and reduced premature cell death in DMD iPSC-CMs. Bioengineered platforms for calcium imaging and Southern blot analysis of telomere restriction fragments were used to assess telomere length, and the study demonstrated that TRF2 preservation of telomeres enhanced cell survival. These findings highlight the importance of iPSCs in not only studying disease mechanisms but also developing therapeutic interventions, suggesting that telomere preservation via TRF2 could hold promise for treating DMD-associated cardiac failure.

### 3.2. EHT

#### 3.2.1. Construction and Mechanical Properties

EHTs involve the creation of three-dimensional structures that replicate the mechanical and electrical characteristics of native heart tissue. These tissues are constructed using cardiomyocytes derived from iPSCs, along with other cardiac cell types, seeded onto biodegradable scaffolds or hydrogels [91]. These materials provide structural support and facilitate tissue organisation. The mechanical properties of EHT can be customised to mimic the stiffness and elasticity of native heart tissue, which is essential for studying how genetic mutations impact the heart’s mechanical function.

Recent advances have focused on incorporating mechanical stimulation into EHTs, allowing researchers to study how genetic mutations affect heart tissue mechanisms under different physiological conditions, such as stretching and contraction [92]. For example, mechanical stretching in EHTs allows researchers to observe how mutations in sarcomere genes, such as *MYH7*, affect contraction and relaxation in inherited cardiomyopathies [93]. These innovations differentiate EHTs from simpler 2D models by enabling the study of heart tissue function under conditions that closely replicate in vivo environments.

#### 3.2.2. Functional Studies in EHT Models

By incorporating sensors and electrodes into EHT, researchers can measure electrical activity and contractile force, offering valuable insights into how mutations influence the electrophysiological and mechanical functions of the heart [92]. A recent study by Wang and colleagues utilised a human EHT model of HCM to replicate key features of the disease [94]. Their EHTs, generated using healthy human cardiac fibroblasts alongside iPSC-CMs carrying a beta-myosin mutation (MYH7-R403Q), exhibited mature cardiac phenotypes with increased tissue size, cell volume and altered sarcomere structures, mimicking those seen in HCM patients. Additionally, the R403Q EHTs exhibited increased twitch amplitude, slower contractile kinetics, prolonged action potential durations, and slower calcium transient decay time—typical findings in myocardial tissue from HCM patients. This study further demonstrated that EHTs provide an excellent platform for testing the mechanical and electrophysiological effects of disease-specific mutations in a controlled setting.

#### 3.2.3. Drug Screening and Therapeutic Testing

EHTs serve as a powerful platform for screening potential drugs and therapeutic agents. By creating patient-specific EHTs using iPSC-derived cells, researchers can evaluate how different drugs affect heart tissue in a physiologically relevant manner.

In the study by Wang and colleagues, the impact of chronic mavacamten treatment—a first-in-class cardiac myosin inhibitor for HCM patients—was investigated [94]. After five weeks of mavacamten treatment, the R403Q EHTs exhibited shortened relaxation time, reduced action potential prolongation, and decreased expression of B-type natriuretic peptide (BNP) mRNA and protein. Additionally, mavacamten treatment led to an increase in sarcomere length and improved sarcomere organisation. Mavacamten has since been identified as a potentially crucial therapy for obstructive HCM patients as an alternative to myomectomy surgery [95]. This highlights how multicellular models like EHTs can facilitate the translation of experimental therapies into clinical practice.

## 4. Insights into Cellular and Molecular Mechanisms

### 4.1. Paracrine Signalling between Cardiac Cells

Cells in the heart communicate through the secretion of signalling molecules, which can influence the behaviour of neighbouring cells. In co-culture systems, researchers can study how mutations affect paracrine signalling pathways, such as those involving growth factors, cytokines, and extracellular vesicles. A study by Kane and colleagues investigated the effect of cardiac fibroblasts on human iPSC-CMs Ca^2+^ cycling. It identified that when human iPSC-CMs are cultured in cardiac fibroblast-conditioned media or in non-contact co-cultures with cardiac fibroblasts, they demonstrated slower Ca^2+^ cycling [96]. This study demonstrates the importance of understanding the key paracrine mediators of cardiac fibroblast crosstalk if we are to better understand the pathological fibrosis that occurs in many IHDs.

### 4.2. Mechanical and Electrical Cell–Cell Interactions

Direct interactions between cardiac cell types, such as the interactions mediated by gap junctions and adherens junctions, are critical for maintaining the structural and functional integrity of the heart, and dysfunction of these interactions has been implicated in IHDs [97]. Co-culture systems allow for the examination of how these interactions are disrupted by genetic mutations. For example, Giacomelli and colleagues identified that human iPSC-CMs in tricellular combinations with human iPSC-derived cardiac stromal cells and cardiac fibroblasts have better contractility and mitochondrial respiration than organoids in the absence of cardiac fibroblasts [98]. They identified that these improvements are through connexin 43 (CX43) gap channels and increased intracellular cyclic AMP. Interestingly, they also identified that organoids containing cardiac fibroblasts derived from patients with arrhythmogenic cardiomyopathy recapitulate features of the disease.

### 4.3. ECM Remodelling

The ECM provides structural support to cardiac cells and regulates their behaviour. In co-culture systems, researchers can investigate how mutations in genes that regulate ECM production or degradation contribute to pathological remodelling, as seen in conditions like myocardial fibrosis. It is well known that ECM proteins interact with cardiomyocytes through the integrin receptors found on the cardiomyocytes [99]. A study investigating the role that ECM proteins play in cardiac remodelling identified that the integrin-binding sequence of arginine-glycine-aspartic acid induces recruitment of the sarcoplasmic reticulum for Ca^2+^ cycling in excitation–contraction coupling in cardiomyocytes [100]. The flanking sequence around this tripeptide integrin-binding sequence determines affinity for integrins. Integrin ligand–receptor interactions in the in vitro setting, reflecting the interactions between ECM proteins and cardiomyocytes, have been found to modulate cardiomyocyte Ca^2+^ cycling [101]. This has important implications in our understanding of pathophysiology of IHDs and therapeutic strategies that can correct the Ca^2+^ cycling dysfunction often found in cardiomyopathies.

### 4.4. Metabolic Coupling in the Heart

The heart is a highly metabolically active organ, and the metabolic interactions between different cell types are crucial for maintaining cardiac function. Barth syndrome, an x-linked cardiac and skeletal myopathy, results from a mutation in the gene tafazzin [101]. Tafazzin is responsible for the production of the enzyme acyltransferase. Reduced acyltranferase function leads to decreased acetylation of cardiolipin—the major phospholipid of the mitochondrial inner membrane [102]. By using cas9-mediated genome editing of cardiomyocytes derived from patients, it has been shown that a mutation in tafazzin can recapitulate the patient disease phenotype in wild-type cells [103]. Tafazzin deficiency increased the production of reactive oxygen species (ROS), and suppression of ROS ameliorates the metabolic dysfunction in Barth syndrome human iPSC-CMs, demonstrating the critical role of metabolic regulation in the disease.

## 5. Limitations and Future Directions

### 5.1. Limitations and Challenges

The potential applications of human iPSC-CMs in both research and therapy are significant. However, it is essential to address the immaturity of human iPSC-CM structures [104]. These cells exhibit structural characteristics more like neonatal or embryonic cardiomyocytes than those of adult cardiomyocytes (Table 2). Typically, human iPSC-CMs are large, flat, round, and possess a single nucleus, though they can adapt considerably to their culture environment [105,106]. They generally mimic the phenotype of human cardiomyocytes during the early stages of cardiogenesis. At this developmental phase, the myocardium has not yet transitioned from hyperplastic to physiological hypertrophic growth. In physiological hypertrophic growth, elongated, rod-like cardiomyocytes align anisotropically and connect electrically via unidirectional gap junctions, facilitating efficient contraction of the cardiomyocyte syncytium in the developing heart [107]. However, this directional propagation of cardiomyocyte excitation is not observed in spontaneously beating human iPSC-CMs in vitro, likely due to the absence of extracellular stimuli found in the developing heart [108].

One significant limitation of human iPSC-CMs is their restricted contractility, which is attributed to the immaturity of their sarcomere structure [109,110]. Sarcomeres, defined as the distance between two Z-lines, are crucial for force production. In adult cardiomyocytes, the optimal sarcomere length for maximum contractile force is 2.2 μm under loaded conditions and 1.8 μm under unloaded conditions [111]. In contrast, human iPSC-CMs possess shorter (1.6 μm) and disorganised sarcomeres, akin to those in fetal cardiomyocytes or in the pathological state [112]. Studies have shown that human iPSC-CMs primarily exhibit Z-disk and I-band structures, although these characteristics can vary significantly depending on the seeding conditions and extracellular interactions [113]. Despite variations in human iPSC-CMs phenotypes across different studies, it is generally agreed that these cells produce much less force than healthy adult cardiomyocytes [114].

Additionally, human iPSC-CMs exhibit suboptimal mitochondrial maturation. In adult cardiomyocytes, mitochondrial biogenesis significantly increases mitochondrial content to meet higher energetic demands [115]. In adult ventricular myocytes, mitochondria occupy about 30% of the total cell volume, compared to just 2% in human iPSC-CMs [116]. Moreover, mitochondria in human iPSC-CMs have lower activity due to immature cristae on their inner membrane [116,117]. Unlike healthy adult cardiomyocytes, which primarily rely on fatty acid oxidation for energy, human iPSC-CMs, like the foetal heart, heavily depend on glycolysis [118].

It has been observed that many ultrastructural features of human iPSC-CMs maturation develop with prolonged culture. Increases in nucleation, myofibril density, and expression of contractile proteins such as β-myosin heavy chain have been reported in cultures 120–360 days post-differentiation [113,119]. However, it remains unclear whether these developments are due to genuine maturation over time or result from senescence caused by the culture conditions.

### 5.2. Future Directions in IHD Research

The use of human iPSCs in investigating IHDs has significantly advanced our understanding of the molecular and cellular mechanisms underlying these conditions. However, there are still several key areas where future research could further enhance the field. Recent bioengineering strategies and organ-on-a-chip technologies offer promising solutions to these limitations, pushing the boundaries of iPSC-based cardiac models.

A key strategy in addressing the immaturity of iPSC-CMs has been the use of bioengineering approaches including mechanical and electrical conditioning. Studies have demonstrated that applying cyclic mechanical stretching and electrical stimulation to iPSC-CMs can significantly enhance their maturation by promoting more adult-like structural and functional properties, including improved contractility, alignment, and electrophysiological behaviour [120]. These strategies help overcome the developmental immaturity of iPSC-CMs, allowing for more physiologically relevant disease models that closely mimic adult heart function.

Additionally, the integration of iPSC-CMs into three-dimensional bioengineered tissues, such as EHTs and organoids, has provided more accurate representation of the multicellular environment found in the human heart. These systems incorporate cardiomyocytes together with non-cardiomyocyte components of the myocardium to better replicate the complex cell–cell interactions and mechanical properties of the myocardium. In particular, organ-on-a-chip technologies have recently emerged as a powerful tool to model cardiac diseases, offering the ability to simulate key physiological and mechanical conditions such as fluid flow, mechanical strain, and electrical stimulation [121]. This allows researchers to investigate the effects of genetic mutations, drug responses, and tissue remodelling in a highly controlled and reproducible environment.

Recent advances in microfluidic organ-on-a-chip platforms have further enhanced the maturity and functionality of iPSC-CMs. By mimicking the dynamic mechanical forces of the heart, such as pulsatile flow and cyclical stretching, these platforms promote the maturation of iPSC-CMs into more physiologically relevant cardiomyocytes with improved sarcomere organisation, calcium handling, and contractile properties. This progress holds great potential for advancing our understanding of IHDs, particularly in relation to how genetic mutations and environmental factors impact heart development and disease progression.

Another promising direction is the integration of human iPSC technology with advanced gene-editing tools such as CRISPR/Cas9. This combination allows for the precise modelling of genetic mutations associated with IHDs, enabling researchers to study not only the impact of specific mutations but also the potential for correcting them at the genetic level. By correcting mutations in vitro, researchers can study the effects of genetic restoration on cellular function and explore the potential for gene-based therapies to treat IHDs.

Furthermore, the integration of human iPSC-derived cardiac models with high-throughput screening technologies holds significant potential. This approach could enable large-scale screening of genetic variants and environmental factors that contribute to IHDs, as well as the identification of novel biomarkers and therapeutic targets.

In addition, as artificial intelligence and machine learning algorithms become more sophisticated, there is growing potential to leverage large datasets generated from iPSC-derived cardiac models to identify pattern and predict disease outcomes. These technologies could enable more accurate predictions of disease progression, drug efficacy, and patient responses to therapies, paving the way for precision medicine in the treatment of inherited heart diseases.

Finally, the exploration of patient-specific human iPSC models will continue to be a critical focus in advancing personalised medicine. By generating human iPSCs from diverse populations, including those with rare IHD mutations, researchers can better understand the variability in disease presentation and response to treatments. This personalised approach to IHD research has the potential to revolutionise the way we diagnose, study, and ultimately treat inherited heart diseases.

## 6. Conclusions

The development of stem cell-derived models has revolutionised the study of inherited heart diseases, providing unprecedented insights into the molecular and cellular mechanisms underlying these conditions. Single-cell models using human iPSC-CMs allow researchers to study the effects of genetic mutations at a cellular level, offering valuable information on the roles of specific genes and proteins in disease pathogenesis. Genome editing technologies, such as CRISPR/Cas9, further enhance the ability to investigate the genetic underpinnings of IHDs, enabling precise manipulation of specific genes and mutations. In addition, multicellular models and tissue engineering approaches, such as cardiac organoids, engineered heart tissue, and co-culture systems, provide a more comprehensive and physiologically relevant platform to study disease mechanisms. These models allow researchers to investigate the complex interactions between different cell types within the heart, providing deeper insights into the pathophysiology of IHDs and enabling the development of more effective diagnostic and therapeutic strategies. By leveraging these innovative models, researchers can gain a deeper understanding of the molecular and cellular mechanisms underlying IHDs, ultimately paving the way for more effective diagnostic and therapeutic strategies.

## Figures and Tables

**Table 1:** Available human induced pluripotent stem cell-derived cells used in disease modelling. CM = cardiomyocyte, EC = endothelial cell, RBC = red blood cell, SMC = smooth muscle cell.

Pathology	Cell Type Involved	Mutation	(Drug/Treatment) Test	Ref.
Cardiomyocytes				
Barth syndrome	CM	Tafazzin	Genetic rescue	[35]
Brugada syndrome	CM	SCN5A-1795insD mutation	Mexiletine	[36,37]
Catecholaminergic polymorphic ventricular tachycardia type 1	CM	Ryanodine receptor 2 (RYR2)	Isoproterenol	[38,39]
Familial hypertrophic cardiomyopathy	CM	MYH7 Arg663His	Verapamil, diltiazem, mexiletine among 15 drugs	[40]
Hypoplastic left heart syndrome	CM	Patient-derived (GM12601)	Isoproterenol	[41]
Ischaemic heart damage	CM	Aldehyde dehydrogenase 2 (ALDH-2) deficiency	siRNA knockdown	[42]
LEOPARD syndrome	CM and 3 germ layers	PTPN11	Rapamycin	[43]
LQT1,2,3,5,8,14	CM	Patient-derived	Common drugs	[44,45,46,47,48,49]
Endothelial				
Healthy	EC	N/A	Flow-induced disease and simvastatin	[50,51]
Hutchison-Gilford Progeria Syndrome	EC	Patient-derived	N/A	[52]
Lymphocytes				
Healthy	B-cell lymphoid lineage	N/A	N/A	[53]
Red blood cells				
Health	CM and RBC	N/A	Toxicity of RBC	[54]
Sickle	RBC	Patient-derived	Biallelic correction	[55]
Smooth muscle cells				
Supravalvular aortic stenosis	SMC	Elastin (ELN)	Elastin recombinant protein	[56]
Marfan syndrome	SMC	FBN1	Gene editing	[57]

**Table 2:** Structural, electrophysiological, and molecular marker differences between immature and mature cardiomyocytes. − signifies absence of marker and + signifies presence of marker.

		Immature CMs	Mature CMs
Structure			
Shape		Irregular	Rod-shaped
Area		~480 µm^2^	~1700 µm^2^
Volume		Small	Large
Sarcomere organisation		Disorganised and less developed	Organised and M-line developed
Mitochondrial population		Few	Abundant
T-tubule organisation		Absent	Scarce in atrial, abundant in ventricular
Glucose metabolism		High	Low
Nucleus morphology		Mononuclear	Mononuclear, binuclear, multinuclear
Electrophysiology			
Spontaneous activity		Very frequent	Absent
Maximum diastolic potential		−60 mV	−70mV (atrial) to −80 mV (ventricular)
Maximum upstroke velocity		44–50 V/s	200 V/s
Action potential amplitude		94–113 mV	80–130 mV
* Action potential duration at 50%		60–130 ms	200 ms (atrial), 200–300 ms (ventricular)
* Action potential duration at 90%		80–160 ms	200–400 ms
Force generation		100–150 Pa for a single cell	Myocardium tensile force ≈ 56 kPa
Elastic modulus		466 Pa	22–55 kPa
Molecular markers			
Gap junction	Cx40	−	+(atrial), −(ventricular)
	Cx43	+	+
	Cx45	+	−
Ion channel	KCNA5	+	+(atrial), −(ventricular)
	NCX1	+	+
	SERCA2a	+	+
	RYR2	+	+
	Ca_v_ 1.2	+	+
	K_ir_ 2.1	+	+
	K_v_ 4.3	+	+
	KChip 2	+	+
	KCNH2 (HERG)	+	+
Structural protein	TNNT2	+	+
	ACTN2	+	+
	MLC2A	+	+
	MLC2V	+	−(atrial), +(ventricular)
	MYL2	+	+
	MYH6	+	+
Master gene	NKX2.5	+	±

* Action potential duration for human iPSC-CMs depends on seeding conditions [106].

## Abbreviations

ALDH-2	Aldehyde dehydrogenase 2
ARVC	Arrhythmogenic right ventricular cardiomyopathy
BNP	B-type natriuretic peptide
CX43	Connexin 43
DCM	Dilated cardiomyopathy
DMD	Duchenne Muscular Dystrophy
DSB	Double-strand break
EC	Endothelial cell
EHT	Engineered heart tissue
ELN	Elastin
ESC	Embryonic stem cells
GSK	Glycogen synthase kinase
HCM	Hypertrophic cardiomyopathy
IHD	Inherited heart disease
iPSC	Induced pluripotent stem cells
iPSC-CM	induced pluripotent stem cell-derived cardiomyocyte
LQTS	Long QT syndrome
NHEJ	Nonhomologous end-joining
RBC	Red blood cell
ROS	Reactive oxygen species
RYR2	Ryanodine receptor 2
SMC	Smooth muscle cell
SQTS	Short QT syndrome
VUS	Variant of uncertain significance
